# Radar Sensor Signal Deinterleaving Through the Use of a Hypergeometrical Divide Algorithm Adapted to Pulse Clustering

**DOI:** 10.3390/s26123817

**Published:** 2026-06-16

**Authors:** Łukasz Rybak, Janusz Dudczyk, Jakub Olszewski

**Affiliations:** Institute of Communications Systems, Faculty of Electronics, Military University of Technology, 00-908 Warsaw, Poland; lukasz.rybak@wat.edu.pl (Ł.R.); jakub.olszewski01@student.wat.edu.pl (J.O.)

**Keywords:** clustering, data particle, deinterleaving, hypergeometrical divide

## Abstract

Deinterleaving of radar signals is an important issue in processing radiolocation data as a part of the analysis of complex electromagnetic signal environments. The introduction contains a draft of radiolocation signal deinterleaving. The authors began with a high-level view of contemporary challenges in radar signal processing and concluded with the genesis of the signals deinterleaving problem and its technical details. A chronicle of the Hypergeometrical Divide (HypGD) algorithm, describing important stages of its development, and a synthesis of knowledge from scientific reports on the examined method were also presented. The aim of this article is to define the performance of the HypGD algorithm adapted to the deinterleaving of radiolocation signals. The research focused on evaluating the clustering performance of the adapted HypGD algorithm, including its ability to determine groups corresponding to emission source types and to support the analysis of radar pulses in complex signal environments. The authors referred to recent publications in the field of radar signal deinterleaving to systematize the current state of knowledge in this area. A detailed, systematic review of significant works on the HypGD method and its applications was provided. The research used real, anonymized data. The results allowed the formulation of conclusions that contribute to the current state of knowledge. For the first time, the effectiveness of the HypGD algorithm adapted to the deinterleaving of radiolocation signals through pulse clustering has been demonstrated.

## 1. Introduction

Performing tasks in the cyber domain [1] requires rapid adaptation to keep pace with the dynamic development of technology. The proliferation of unmanned systems, especially those operating in airspace, implies a growing number of drone incidents in many countries around the world [2]. As a consequence of this challenge, there is a growing demand for counter-drone systems, whose essential components are radar devices [2,3]. Therefore, it can be seen that the popularization of the use of unmanned platforms in operations has posed significant new challenges in modern signal analysis [4]. One of these is the area of detection, targeting, and tracking of unmanned aerial vehicles [5,6]. The second issue is their use as carriers of various types of sensors, including electronic intelligence (ELINT) devices [7], for the purpose of conducting reconnaissance to build an information advantage [8]. Another crucial area in modern signal analysis is conducting electronic reconnaissance of anti-drone systems, often using multiple radar devices of different or the same type.

Processing radar signals, as well as recognizing, classifying, and identifying their emission sources, are key tasks performed during operations in the modern environment. With the ongoing development of security systems, including radar systems, their users utilize numerous types of devices emitting electromagnetic energy. One group of such equipment includes radars, which perform tasks related to detection, tracking, classification, and identification of objects. An area directly related to the operation of radar systems is electronic intelligence, abbreviated as ELINT. ELINT systems are passive solutions that analyze signals across a wide frequency band, correlate signal characteristics with radar operating frequencies ranging from 0.5 to 18 GHz [9], and perform tasks related to the detection, identification, and classification of electromagnetic radiation sources [10]. They constitute a crucial tool for building situational awareness and gaining an information advantage in the modern environment. This is accomplished by supporting the process of assessing potential threats, developing information on the location of emission sources within the area of operations, and, as a result, taking immediate action to neutralize potential threats. This highlights the fact that ELINT systems are essential components of modern environments and constitute an integral part of advanced signal monitoring and analysis systems.

The basic object processed in these systems is a data structure describing a single pulse, known as a PDW (Pulse Description Word). This is a vector of characteristic features that describes in detail the parameters of a single radar pulse. Time of Arrival (TOA), Pulse Repetition Interval (PRI), Pulse Width (PW), Radio Frequency (RF), and Amplitude (AMP) [11] are the key pulse descriptors mentioned in the literature, the set of which allows for determining the technical parameters of the analyzed emission source. During the individual stages of ELINT data processing, a multidimensional data set composed of multiple PDW vectors enables the identification of the operational characteristics of electromagnetic radiation sources recorded during intelligence operations.

Given the abundance of electromagnetic radiation sources in the modern environment, the process of deinterleaving radar signals poses a significant challenge for ELINT systems. This is the stage of ELINT data processing in which the aforementioned pulses are referred to as interleaved. This should be understood as a situation in which the number of emission sources is unknown, and there is no information about the assignment of individual pulses to specific radars. This situation serves as the starting point for the next stage of signal intelligence data processing: signals deinterleaving [12]. From a high-level perspective, this problem focuses on processing pulse parameters with two goals in mind. The first is to precisely determine the number of emission sources associated with the recorded signals. The second is the unambiguous identification of the emission source for each collected pulse, which is described by a one-to-many cardinality relationship, according to the database representation from the emission source’s perspective.

Machine learning is currently one of the key elements used for this purpose in ELINT systems. Traditional processing of such signals across numerous stages relies on experts manually performing specialized analyses at the level of particular pulses, comparing them against a database of patterns. This process can be time-consuming, and its automation is non-trivial, inter alia, due to the fact that processing involves multidimensional data sets requiring detailed analysis. Therefore, it is still reasonable to attempt to apply artificial intelligence algorithms to electronic intelligence data processing, including the analysis of PDW data sets [13]. In particular, supervised and unsupervised machine learning methods are of great importance in this area.

This article covers radar signals deinterleaving and directs research towards unsupervised learning methods, which constitute a branch of machine learning. A key issue in this area is clustering, a topic frequently explored by researchers in the international literature. A distinctive feature of this area is the use of datasets containing objects to which no labels have been assigned [14]. The use of clustering algorithms in processing such databases allows for the extraction of patterns by analyzing hidden relationships in the distributions of individual attributes, which is a key aspect of radar signals deinterleaving. It is worth noting that identifying the optimal method for implementing this process is currently challenging.

The subject of this article covers the adaptation of the data particle hypergeometrical divide algorithm to the task of data clustering, which, in the context of radiolocation signal processing, can be identified with the process of deinterleaving these signals. The aforementioned method is an extension of earlier work on gravitational classification models and of the Geometrical Divide algorithm initiated in 2020 [15]. In subsequent studies, this method was developed and modified, resulting in the establishment of its variant—Unequal Geometrical Divide (UGD) [16]. The resulting analysis of its properties formed the basis for the evaluation of this approach, which gave rise to the creation of a hypergeometric data particle divide model, enabling the classification of multidimensional data sets [17]. The new method, HypGD, developed by one of the authors of the above article and this one, has been applied to the analysis of radar data, in particular in the process of identifying signals originating from multiple emitters of the same type [10]. An important measure determining the quality of an algorithm’s performance is its efficiency. Therefore, one of the works examined the influence of the data particle depth level on the effect of the only parameter required during the implementation of the HypGD algorithm [18]. The results and conclusions from the aforementioned research formed the basis for further work, enabling the demonstration of additional properties of the data particle hypergeometrical divide algorithm.

Based on the facts presented in the previous paragraphs, it is worth emphasizing that, to date, no attempt has been made in the international literature to apply the HypGD algorithm to dataset clustering. Therefore, the aim of this paper was to determine the performance of the data particle hypergeometrical divide algorithm for deinterleaving radar signals. In particular, it assessed the accuracy of determining the number of emission source types and the precision of individual pulse labels.

## 2. Literature Review

The deinterleaving of radar signals is a key stage of data analysis in modern electronic intelligence systems. The goal of this process is to separate overlapping signals from multiple emission sources and to enable further processing for the classification and identification of the devices from which those pulses originate. Due to the multifunctionality and agility of modern radar devices, this task is not trivial [19]. Its implementation requires the development of advanced methods for signals analysis and processing. Numerous works devoted to the issue of deinterleaving have appeared in the global scientific literature over the last dozen months. These papers introduce entirely new approaches based on artificial intelligence—deep learning models and unsupervised machine learning methods. However, the authors of this article have not found any works in the global literature that attempt to use a data-gravitational model for radar signals deinterleaving. A synthetic summary of modern methods of radar signals deinterleaving is presented at the end of Section 2.1.

### 2.1. Modern Methods of Radar Signals Deinterleaving

In 2024, Kocamış M.B. et al. [19] proposed a signals deinterleaving method based on image analysis and processing. The approach involved constructing two-dimensional images with a black background, where the width represented the time-of-arrival (TOA) parameter, and the height represented the amplitude (AMP) parameter. Corresponding to the values of PDW pulse parameters collected in the database, particular pixels in the constructed image were filled with white. The resulting radar signal representation was processed by the YOLOv8n algorithm. Synthetic data were used in the research. The described method achieved an mAP50 above 0.98 when processing a dataset with a 30% missing pulse rate.

In the same year, Liu H. et al. [20] developed a multi-parameter deep learning method designed to address complex emission source identification tasks based on a single-pulse description word (PDW). A significant feature of the method is the absence of a parameter defining the time interval between neighboring pulses—the pulse repetition period (PRI). In contrast, it is worth emphasizing that this attribute is often used in solving the described problem. The study was conducted on a synthesized dataset of pulse descriptor words. The results demonstrated superior performance of the method compared to previously published solutions.

Also in 2024, Chen T. et al. [21] proposed a method from the domain of image analysis and processing, which is based on mimetic image mapping of the antenna scan pattern. This solution also implements the SOLOv2 instance segmentation network. The research used a real dataset. For signals with a 30% missing pulse rate, the method is characterized by a 9−24% increase in accuracy compared to classical algorithms and solutions based on YOLOv8 and U-Net networks. It was emphasized that the method is effective in situations with high overlap of signal parameter ranges from different sources and is adaptive to demanding conditions—measurement errors, missing pulses, and spurious pulses.

In the first quarter of 2025, Gunn E. et al. [22] proposed an approach to deinterleaving signals coming from an unknown number of emission sources based on metric learning and a transformer trained with a triplet loss function. Emphasizing the low importance of individual pulses in the problem at hand, the authors pointed out that the described solution uses information about the relationships between pulses in the context of entire sequences. The studies were conducted on synthetic signals coming from 2−20 emission sources. The applied PDW structure consisted of the following attributes: time of arrival, radio frequency, pulse width, angle of arrival, and amplitude. The presented solution outperformed the capabilities of the Gated Recurrent Unit (GRU), a type of Recurrent Neural Network (RNN), and the same model with the HDBSCAN algorithm [23].

In May 2025, Zhang J. et al. [24] proposed a combination of hierarchical clustering based on Kernel Density Estimation–Kullback–Leibler Divergence-Template Matching (KDE-KLD-TM) and a time-frequency convolution network (TFCN) for PDW sorting applied to pulses from different emission sources in dense environments. The pulse repetition interval (PRI) was not used at this stage of data processing. Research was conducted on a real dataset. The system sorted over two million pulses in just over 58 s, achieving a recognition accuracy of over 96%.

In June 2025, Li C. et al. [25] analyzed the problem of deinterleaving radar signals represented by split pulses, which, as mentioned at the time, can be determined by overlapping frequency bands of the devices, different modulation types, and the surrounding propagation space. To solve this problem, Li C. et al. proposed a radar signal deinterleaving and parameter estimation structure and developed a new convolutional bidirectional recurrent neural network (CBRNN). The solution does not reject split pulses but classifies distortion types and estimates the original PDW parameters. The studies were conducted on simulated data. The method enables stable recognition of radar operating modes even in environments with signal degradation.

At the end of 2025, Çağlan Ş. et al. [26] published a paper comparing the properties of the OPTICS algorithm and the *k*-means method. The problem involved the deinterleaving of signals based on carrier frequency and pulse duration from several emission sources. The studies were conducted on a synthetic PDW dataset without missing and spurious pulses. Experiments showed that OPTICS has good properties in the deinterleaving of signals from several emission sources, which, in the space representing the RF and PW dependence, create irregular shapes with different densities. It was also shown that the *k*-means method does not have good properties for this problem. It was indicated that one group may contain pulses from multiple emission sources, which may define the problem of deinterleaving signals from multiple emission sources of the same type, which is an inherent step in the SEI process [10].

The synthetic summary of approaches described above is presented in Table 1.

When summarizing the latest literature, it can be observed that the artificial intelligence methods chosen for signal deinterleaving mostly belong to the deep learning family. However, among these algorithms, researchers sometimes apply approaches dedicated to data clustering. Most recent experiments have used synthetic datasets. However, there are few publications describing research conducted on real datasets concerning radar signals. Most papers on this topic come from China, Türkiye, and the United Kingdom, which is consistent with the data presented in article [27]. In the world literature on radar signal deinterleaving, no attempt has been made to use the Hypergeometrical Divide algorithm for this purpose.

### 2.2. Applications and Properties of Data Particle Geometrical Divide Methods

Gravitational models, based on the law of universal gravitation, have been used since the 1970s [28]. These algorithms allow for the creation of data particles using gravitational divide, which is useful for processing electronic intelligence data. As this philosophy evolved, new methods were developed, expanding the original concept and extending its scope of application. The data particle hypergeometrical divide algorithm is one of these methods. Work on this method began in 2020, when a method called Geometrical Divide was created. This solution is based on the principles of gravitational classification, meaning that each class is represented by a data particle with a specific mass, center of gravity, and label. It enables object classification in the ℝ^2^ dimension feature space [15]. In the Geometrical Divide approach cited here, a data particle is iteratively divided in the feature space along a straight line defined by the centroids, thereby generating smaller subparticles that better generalize the class’s actual shape. The GD method described in this paper was combined with algorithms for defining the mentioned data particle parameters. As a result, the effectiveness of the mentioned algorithm in the classification process was demonstrated. It provided a basis for deeper evaluation and for continuing work on the algorithm in the following years.

In [16], the effectiveness of the Geometrical Divide method and its variant—Unequal Geometrical Divide was compared to the 1CT1P (One Class Template—One Particle) approach. The experiments were conducted on simulated and real imbalanced datasets, containing objects described in the ℝ^2^ feature space. The tests were performed using various particle mass determination algorithms, including SLA (Stochastic Learning Algorithm) [29], BLA (Bath-update Algorithm) [29], and *n*-MM (*n*-Mass Model). The cited works confirmed the effectiveness of GD and UGD in classifying imbalanced datasets. This constituted another step in the development of this family of approaches.

Further development of this group of algorithms included the creation of a method with the acronym HypGD, which derives from its full name—data particle hypergeometrical divide. It has been dedicated to object classification within multidimensional data processing [17]. It is worth noting that the described method, which is an outgrowth of earlier work, enables classification of datasets in an *n*-dimensional feature space, making it significantly more useful than its previous versions. Furthermore, the Hypergeometrical Divide is characterized by a shorter data processing time than the widely known *k*-NN (*k*-Nearest Neighbors) algorithm. However, a disadvantage of HypGD is that it requires setting the d parameter, which specifies the depth of the data particle division, and it must be adjusted individually for a particular data set.

In the article [18], it was demonstrated that by using the technical parameters of the emission source included in the PDW: RF, PD, and AMP, the HypGD algorithm recognizes patterns more efficiently than the compared *k*-NN algorithm. Pulse signals datasets were used in the experiments. Those signals came from anonymized real electronic intelligence signals. The data particle hypergeometrical divide algorithm performs the entire process much faster, which is useful in advanced signal monitoring and analysis systems, where minimizing the processing time is key.

The research described in [19] showed that increasing the level of division depth positively affects the effectiveness of this method, but only up to a certain threshold. Further iterative increases in this value do not improve the classification results. Experiments were then conducted on multi-domain real datasets from publicly available sources. It was concluded that achieving high precision does not require using the maximum level of division depth. It was determined that further research should be directed toward developing a method for defining the division depth value for a data particle.

Similar studies, performed on electronic intelligence data, yielded results consistent with those obtained in previous studies. It was confirmed that increasing the *d* parameter allowed for obtaining better classification results, at the cost of a slightly increased data processing time [30].

To summarize the literature review on the data particle hypergeometrical divide algorithm, it is worth emphasizing that it is positioned within the scope of supervised machine learning techniques. However, published works have repeatedly suggested that further research on this approach could be directed at its applicability to data clustering. The presence of other methods based on the gravitational model within the scope of unsupervised machine learning methods may confirm this thesis.

## 3. Hypergeometrical Divide Algorithm for Clustering and Materials for Its Evaluation

### 3.1. Inherent Features of the Hypergeometrical Divide Algorithm Adapted for the Clustering Task

The adaptation of the Hypergeometrical Divide method for signal deinterleaving is based on the overall idea of the gravitational model of data and the logic, as well as certain assumptions, of a variant of this approach dedicated to data classification. The data particle hypergeometrical divide classifier operates through the creation and analysis of data particles. They represent objects collected in the processed dataset. This method is used to recognize patterns and dependencies in databases, whose objects belong to a feature space of ℝ^2+^ dimension [9,16]. The key assumption is that the data particle is the basic element being processed. It is defined by the vector **p**. The components of this vector are: mass m, center point, and label [31].

In reference to the logic of the data particle hypergeometrical divide method, the entire *n*-element set of input records ***X*** = {**x**_1_, …, **x***_n_*}*^T^*, where **x***_i_* belongs to a *d*-dimensional feature space, is treated at the beginning of clustering as a single data particle. Each particle **p** is geometrically described by two points in the feature space: the center of mass and the center of its geometry. The center of mass is determined as a vector of the arithmetic means for particular attributes of all points belonging to the data particle. The geometric center is defined by a vector whose components are calculated as the middle point of the difference between the minimum and maximum values of particular attributes. Next, a normal vector is determined. It is the difference between the above-mentioned points. If the difference equals zero, then creating additional data particles by dividing is not possible. Based on the normal vector, a dividing hyperplane is created, to which the mass center of the particle belongs [9,16].

The above-described procedures for defining the values of the aforementioned parameters are embedded in the theory of data particle hypergeometrical divide. They were applied for the purpose of developing a method dedicated to the deinterleaving of radar signals in the pioneering research described in this article. The data particle hypergeometrical divide algorithm, adapted to perform data clustering, was named Nonhyperparametric Hypergeometrical Divide (NHyp2GD).

### 3.2. Hypergeometrical Divide Algorithm Adapted for the Clustering Task

Moving on to a detailed description of the operations performed within the proposed adaptation of the Hypergeometrical Divide method, it is worth emphasizing that its significant feature is the elimination of the need to manually define hyperparameters. The process begins by determining the weights of particular attributes, in accordance with Formula (1):

*w_i_* = 2*^i^*, *i* ∈ {0, 1, 2}, (1)

where: *w*_0_—AMP, *w*_1_—PW, *w*_2_—RF.

During data processing, the new algorithm autonomously adapts to the characteristics of the dataset, determining a decision threshold, referred to as *ε*. This parameter serves as a boundary value based on which a decision is made whether to further divide a data particle or to terminate the division process for this particle. To determine the value of *ε*, a preliminary division of the entire data set, initially treated as a single particle **p**, is performed. As a result, two data particles, **p***_A_* and **p***_B_*, are generated, based on which the displacement of their mass centers relative to the mass center of particle **p** is determined. This relationship is described by Formula (2):

*∂_k_* = *||µ*(**p***_k_*) − *µ*(**p**)*||*, *k* ∈ {*A*, *B*}, (2)

where *μ*(**p**) denotes the center of mass of data particle **p**, and *µ*(**p***_k_*) denotes the mass center of the particular data particles created from the realization of the first divide.

The obtained shift values form the basis for determining the threshold *ε*, which is assumed to be the minimum value of these shifts, as described by Equation (3):

*ε* = *min* (*∂_A_*, *∂_B_*). (3)

Adopting the minimum value as the threshold ensures that only data particles exhibiting significant spatial separation from particle **p** are classified as observations requiring further splitting. This operation allows the clustering algorithm to be fully adaptive, eliminating the need to manually set a decision threshold.

Next, according to the HypGD algorithm, a split operation is performed for the two initially created data particles. This division for each of these data particles results in two new data particles—**p***_A_* and **p***_B_*. For each of them, the center of mass offset relative to the base particle is calculated according to Formula (1). The obtained result is compared to the threshold *ε*, which determines whether the analyzed particle becomes the final cluster or is forwarded for further division. This decision is made according to the conditions presented below in Table 2. This process is performed locally for each data particle. This distinguishes the new method from the classic Hypergeometrical Divide algorithm.

To ensure reproducibility of the proposed approach, the implementation details of the NHyp2GD clustering procedure are further specified. The algorithm starts by treating the entire dataset as a single data particle. Each analyzed particle is described by its geometric center and mass center, and the difference between these centers determines the direction of the dividing hyperplane. In the presented implementation, the Euclidean distance metric is used to calculate distances between points and mass centers and to assign final observations to the nearest final mass center.

The recursive division of a data particle is stopped when at least one of the stopping conditions is satisfied. A particle is marked as terminal when the mass center shift of the newly created particle is lower than the threshold ε, when the division produces an empty child particle, or when the particle contains only one observation. Singleton particles are therefore treated as final terminal particles and are not divided further. These conditions prevent the algorithm from generating redundant data particles.

The adopted weighting scheme *w_i_* = 2*^i^* was used to introduce a deterministic and reproducible ordering of the analyzed PDW features. In the considered datasets, the order *w*_0_—AMP, *w*_1_—PW, and *w*_2_—RF reflects the assumed increasing discriminative importance of these attributes for separating radar emission sources. However, the weighting rule is not limited to this particular set of features. For other PDW representations, the same rule may be applied after defining the order of attributes according to their assumed relevance. Thus, the weights should be interpreted as a deterministic feature-prioritization mechanism rather than as manually tuned clustering hyperparameters.

From a computational point of view, a single division level requires processing all observations assigned to the analyzed data particle to calculate the centers and assign observations to one of two child particles. For a dataset containing *n* observations, this operation has linear complexity. Therefore, the basic computational complexity of the recursive division process can be expressed as O(*kn*), where *k* denotes the number of division levels and *n* denotes the number of observations in the dataset.

### 3.3. State-of-the-Art Methods Selected for Comparison for Evaluation of the New Algorithm

To evaluate and verify the properties of the proposed method, comparative studies were conducted. For this purpose, well-known clustering algorithms, frequently cited in the international literature, were selected. From the extensive collection of available cluster analysis methods, two methods representing different branches of clustering approaches were selected.

The first choice was the *k*-means approach. It represents a set of non-hierarchical solutions. This algorithm is often referred to in publications as a centroid algorithm. In the case of its use, the data processing is preceded by defining the value of the *k* parameter. This variable determines the number of center points, and therefore the number of groups that will be generated. Assigning particular points to pre-created *k* groups is based on iterative minimization of the sum of squared distances between points belonging to these groups [32].

The second solution selected for comparison was a method from the set of density-based algorithms—Density-Based Spatial Clustering of Applications with Noise (DBSCAN). It is used in the analysis of irregularly shaped clusters. The assumption assigned to DBSCAN concerns the lack of sphericity of the groups, which distinguishes it from the *k*-means algorithm. Furthermore, elements that outline the created clusters are selected from the set. In the case of DBSCAN, it is crucial to choose appropriate model parameters, such as *ε*, which is the neighborhood radius, and *minPts*, which is the minimum number of elements required to create a cluster [33].

HDBSCAN (Hierarchical Density-Based Spatial Clustering) is another method that belongs to the group of clustering algorithms. It extends the DBSCAN approach by introducing a hierarchical density-based clustering procedure, which allows the detection of groups with arbitrary shapes and varying densities. Compared with DBSCAN, its important advantage is the lack of the need to define a single global neighborhood radius. In most cases, the user defines only the minimum cluster size, while the algorithm determines the final partition on the basis of the hierarchical structure of density-connected observations [34].

The last of the selected methods was AGNES (AGglomerative NESting), which represents the agglomerative approach to hierarchical clustering. The algorithm starts by treating each observation as an individual cluster. Then, in subsequent iterations, the closest groups are gradually merged according to the adopted distance measure and linkage criterion. This process leads to the creation of a hierarchical structure of clusters, from which the final partition is obtained by defining the expected number of groups or selecting an appropriate level of the hierarchy [35].

In contrast to the aforementioned methods, in the proposed NHyp2GD approach, all key parameters constitute the model definition, and their values are determined autonomously. These parameters are determined by the characteristics of the analyzed dataset. The algorithms requiring parameterization were implemented as shown in Table 3.

The parameterization presented in Table 3 was empirical and exploratory in nature. The values used were selected after multiple iterations of testing and visual verification of the clustering results obtained. This was due to the fact that the direct application of standard parameter selection procedures, such as silhouette maximization, the elbow method, or the k-distance plot, in some cases led to an incorrect number of clusters and thus to the formation of groups inconsistent with the actual dataset. Therefore, the final parameter values were selected based on numerous experimental iterations, and the selected parameters reflected the number of groups created by the reference algorithms in the most accurate manner.

### 3.4. The Properties of the Applied Dataset

**Figure 1:**
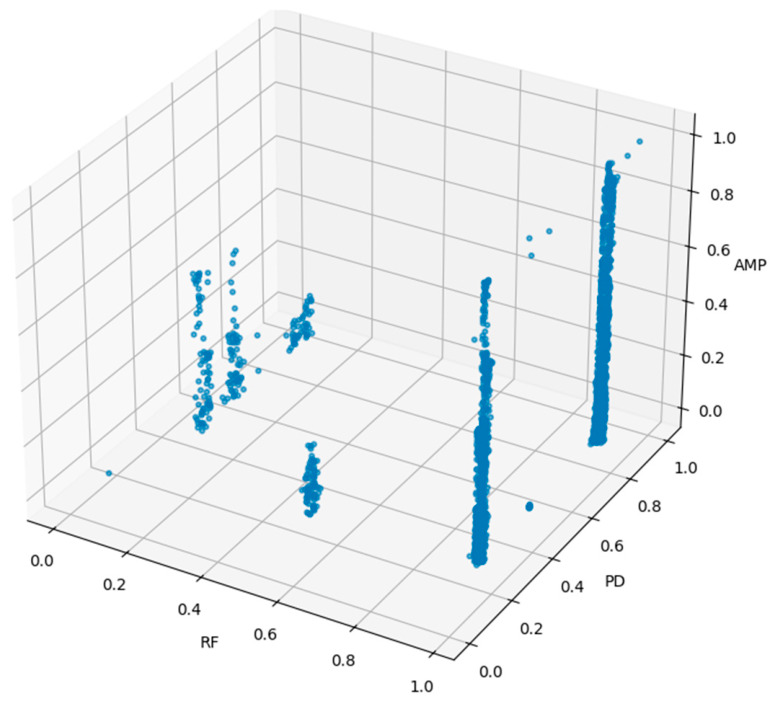
Visualization of the dataset used, each blue point refers to the single pulse (source: own elaboration).

A real dataset was used in the experiments. The collected records described pulses originating from four different modes of three radar emitters. The database used contained 2016 PDW vectors. Each vector was described by three pulse attributes. The set of features analyzed from the processed signals consisted of carrier frequency (FREQ), pulse width (PW), and amplitude (AMP). To ensure that the order of magnitude of the attribute value sets did not influence their significance, the dataset was normalized. For this purpose, the value range of each feature was independently mapped to the range [0, 1]. To evaluate the properties of the developed method in the context of signal deinterleaving, all objects were stripped of information about the label describing their emission source. This was achieved by using a single color for all points. Figure 1 provides a visualization of the created three-dimensional feature space containing elements collected in the dataset.

### 3.5. Clustering Quality Metrics Applied for Deinterleaving Evaluation

In order to evaluate the selected data clustering methods in terms of their application for radar signals deinterleaving, the well-known quality measures presented in Table 4 were used in the experiments. They allowed for the representation of the quality of clustering results based solely on the geometry of the defined clusters.

The Silhouette Score measures the internal cohesion of a cluster and its separation from other clusters. For each point *i*, the Silhouette Score value is determined according to Formula (4):

*silhouette_score*(*i*) = (*b*(*i*) − *a*(*i*)) ∙ (*max*{*a*(*i*), *b*(*i*)})^−1^, (4)

where *a*(*i*) is the average distance of the point from other points belonging to the same cluster, and *b*(*i*) is the average distance of *i* to the points of the nearest neighboring cluster. The determined measure takes values in the range [−1, 1], where values close to 1 indicate well-separated and coherent clusters [36].

The Dunn Index measures the ratio of the minimum distance between clusters to the maximum intra-cluster distance, based on Equation (5):

*dunn_index*(*i*) = *min*_1≤*i*≤*j*≤*m*_ ∂(*C_i_*, *C_j_*) ∙ (*max*_1≤*k*≤*m*_ ∆*_k_*)^−1^, (5)

where *m* defines the number of clusters in the data, and *∂*(*C_i_*, *C_j_*) denotes the inter-cluster distance between *C_i_* and *C_j_*, while ∆*_k_* is the maximum distance between a pair of points within a particular cluster. A higher Dunn Index value indicates better separation of clusters and their greater compactness [37].

## 4. Results

The research was divided into two experiments. The first involved a comparative properties analysis of selected methods. The performance results for specific algorithms are presented in Table 5 as quality measures described in Section 3.5 and data processing times. The best results in particular criterions are in bold.

The proposed NHyp2GD method achieved a Silhouette Score of 0.5128, compared to 0.5107 for the DBSCAN algorithm, 0.5101 for HDBSCAN, slightly lower than AGNES (0.5267), and lower than *k*-means (0.5757). This means that the clusters generated by the developed method are characterized by high internal consistency and good separation from other clusters. A much more pronounced advantage is evident compared with *k*-means and AGNES for the Dunn Index, for which NHyp2GD achieved a value of 0.1844. This is 50 times higher than the *k*-means algorithm used for comparison (0.0035) and almost 29 times higher than AGNES (0.0064), while remaining lower than DBSCAN (0.6628) and HDBSCAN (0.2945). Comparing the clustering execution time, the proposed method completed the data clustering process in 0.0118 s. This was the shortest time among all the analyzed algorithms. NHyp2GD was approximately 2.3 times faster than DBSCAN, which required 0.0274 s, approximately 2.2 times faster than HDBSCAN, which required 0.0256 s, 5 times faster than AGNES (0.0594 s), and 154 times faster than *k*-means (1.8195 s).

**Figure 2:**
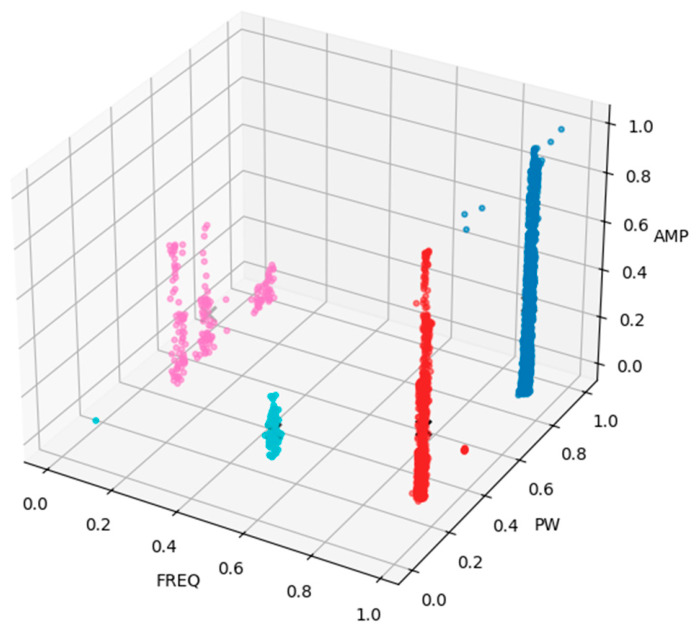
Groups created using NHyp2GD, the “X” symbols overlapped by points refer to centroids of groups, they are auxiliary symbols (source: own elaboration).

The second experiment involved performing an in-depth analysis of the generated feature spaces and the element groups within them, which were created by the particular algorithms. Using the data particle hypergeometrical divide approach in cluster analysis, the NHyp2GD algorithm created four distinct groups, marked in Figure 2 with the following colors: red, purple, blue, and aqua. There were minor deviations in the accuracy of assigning specific particles to groups. They did not have a significantly negative impact on the final clustering results. This is confirmed by the values obtained for the measures used, presented in Table 4.

**Figure 3:**
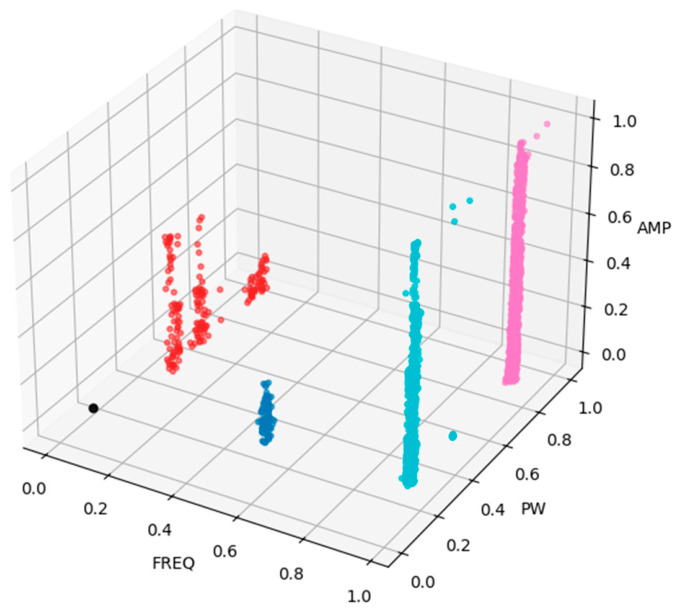
Groups created using DBSCAN (source: own elaboration).

The second feature space analyzed was the one created using an algorithm from the density-based approach branch—DBSCAN. In this case, four clusters were identified, as visualized in Figure 3, they are presented by points in four colors: red, purple, blue and aqua. Single points were marked in dark blue as noise. Compared to the other tested approach—*k*-means, DBSCAN resulted in a few incorrect group assignments. The DBSCAN method created clusters similar to those defined using the NHyp2GD approach. A significant feature of the DBSCAN algorithm, distinguishing it in this comparison, was its ability to identify noise points, i.e., single elements of the feature space located away from high-density areas.

**Figure 4:**
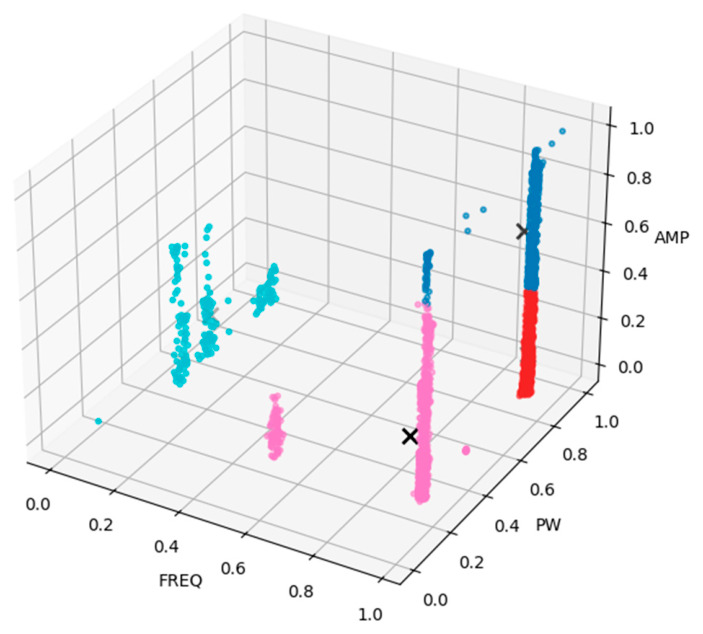
Groups created using *k*-means, the “X” symbols overlapped by points refer to centroids of groups, they are auxiliary symbols (source: own elaboration).

The feature space (Figure 4), which was processed for in-depth analysis, was the result of the *k*-means algorithm. According to the initially defined parameter value defining the number of generated clusters, the *k*-means approach created four clusters of points assigned by the following colors: red, purple, blue and aqua. It is worth noting that the generated image revealed contamination during the clustering process. Particular points from clearly defined clusters were intermingled with neighboring clusters. As a result, the created clusters were characterized by heterogeneity.

**Figure 5:**
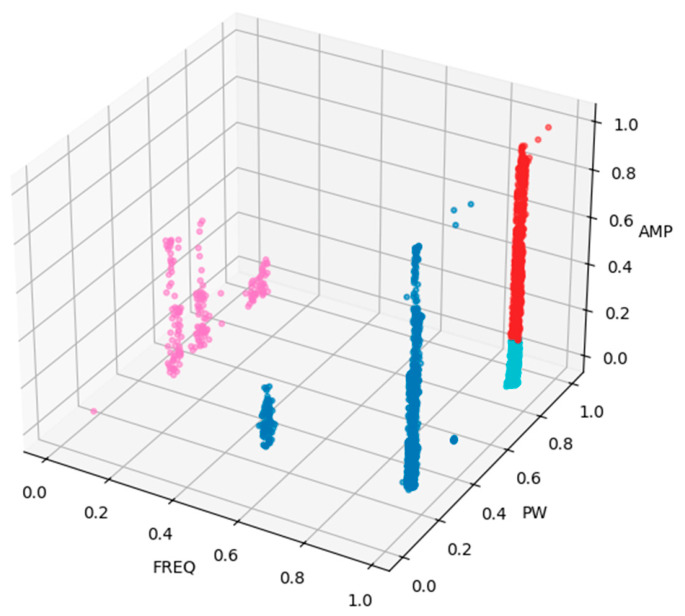
Groups created using AGNES (source: own elaboration).

The feature space presented in Figure 5 shows the result of clustering performed using the AGNES algorithm. Although the method generated the assumed number of four clusters represented by points in four colors: red, purple, blue and aqua. The obtained partition was not fully consistent with the actual cluster distribution. The vertical structure located on the right side of the plot was divided into two separate clusters, despite forming a spatially coherent group. In addition, the blue cluster included not only the main vertical structure, but also a neighboring concentration of points that, given its location in the feature space, should be interpreted as a separate group.

**Figure 6:**
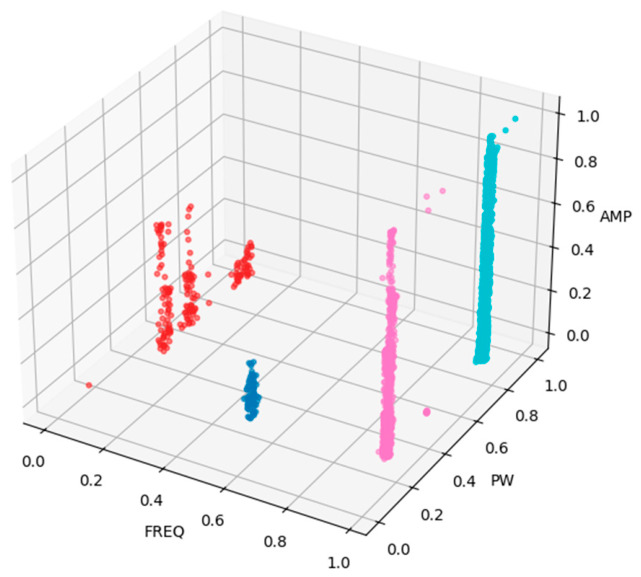
Groups created using HDBSCAN (source: own elaboration).

The feature space presented in Figure 6 illustrates the clustering result obtained using the HDBSCAN algorithm. Created clusters were distinguished by points in four colors: red, purple, blue and aqua. The generated partition reflects the visible spatial distribution of observations in the analyzed space. The algorithm separated the main groups of points into coherent clusters, including the vertical structures located in different regions of the feature space. The obtained result indicates that HDBSCAN was able to reproduce the main structure of the analyzed dataset and assign observations consistently with their spatial arrangement. However, it should be noted that individual observations were not clearly separated as noise but were instead assigned to the generated clusters.

## 5. Conclusions

In this research work, the Hypergeometrical Divide (HypGD) classification algorithm was adapted for data clustering. Demonstrating the strong correlation between cluster analysis from the artificial intelligence domain and radar signal deinterleaving, which is relevant to activities in an electronic environment, the properties of a new variant of this method—Nonhyperparametric Hypergeometrical Divide (NHyp2GD)—were verified. The obtained results demonstrated that the proposed NHyp2GD approach is an effective alternative to established clustering methods, such as the other compared algorithms. Furthermore, the designed solution was confirmed to be efficient when applied to the deinterleaving of radar signals at the level of individual pulses represented in a three-dimensional feature space.

Analysis of the data presented in Table 5, showing the values of quality metrics achieved by the used algorithms, and the visualizations presented in Figure 2, Figure 3, Figure 4, Figure 5 and Figure 6, showed that for the processed dataset, containing data from four modes of three different types of emission sources, the NHyp2GD method achieved good results, reaching the third-highest values for the Silhouette Score and Dunn Index metrics. Furthermore, the pulse grouping process time using the developed NHyp2GD algorithm was shorter than the time required to process the data using the DBSCAN, *k*-means, AGNES, and HDBSCAN approaches. This demonstrates the properties of the developed solution in the context of two-criterion optimization of the signal deinterleaving process—minimizing data processing time and maximizing its efficiency. These objective functions constitute a frequently defined goal in the field of algorithm design.

At the same time, it should be emphasized that the metric values obtained by NHyp2GD remain close to the results achieved by the reference algorithms. Although the achieved results also show that NHyp2GD is not an ideal solution for all analyzed datasets, especially in cases where weaker cluster separation may occur, the developed approach has an important advantage over the compared methods because it does not require prior parameterization before the clustering process is performed. This constitutes a significant advantage of NHyp2GD in the context of its application in radar signal deinterleaving. It eliminates the need for prior knowledge of the number of sources recorded by the ELINT sensor. In the practical context of activities in a modern electronic environment, this information is often unavailable at this stage of electronic intelligence.

The review of the international literature and the research results presented in this article expand the knowledge of data particle hypergeometrical divide algorithms and their applications in electronic intelligence. To date, the methods from the Hypergeometrical Divide family have been successfully used in the deinterleaving of radar signals, classification, and identification of their emission sources. Therefore, it is worth considering applying this type of approach to other stages of ELINT data processing, an example of which is the extraction of the main lobes of recorded signals. Expanding the range of applications may, in the future, lead to the development of a concept for a comprehensive electronic intelligence data processing algorithm.

Considering the issues for further research, it is worth noting that all literature reports on the use of data particle hypergeometrical divide methods in ELINT data processing have used datasets describing signals originating from only a few emission sources. Therefore, it is also a challenge to conduct research using datasets containing more than a dozen emission sources. Future studies should also extend the analysis to higher-dimensional feature spaces, including descriptors such as TOA and DOA, provided that such data can be obtained from subsequent measurement campaigns. Further work should also include the preparation of a separate experimental framework enabling the comparison of NHyp2GD with selected deep learning-based methods used in radar signal deinterleaving, taking into account the different methodological assumptions of these approaches. Another important direction for further development is the extension of NHyp2GD with a mechanism for noise and outlier identification. Such a modification could increase the sensitivity of the methods to local data structures, improve cluster separation, and reduce the influence of disturbing points on the calculation of mass centers and subsequent particle divisions.

## Figures and Tables

**Table 1:** Modern methods of radar signals deinterleaving synthetic summary (source: own elaboration based on [19,20,21,22,24,25,26]).

Author/Year	Country	Method	Method Family	PDW Attributes	Data Type	No. Emitters	Spurious Pulses
Kocamış M.B. et al., 2024 [19]	Türkiye	YOLOv8	Deep Learning	AMP, TOA	Synthetic	1–5	Yes (30% missing pulses)
Liu H. et al., 2024 [20]	China	Transformer (PulseFormer)	Deep Learning	DOA, RF, PA, PW	Synthetic	Not specified	Yes
Chen T. et al., 2024 [21]	China	Pattern mapping + SOLOv2	Deep Learning	PA, PW, RF, TOA	Real	5	Yes (30% missing pulses)
Gunn E. et al., 2025 [22]	United Kingdom	Transformer + Metric Learning	Deep Learning	AOA, AMP, PW, RF, TOA	Synthetic	2–20	YES (handles missing pulses, noise, agile radars)
Zhang J. et al., 2025 [24]	China	KDE-KLD-TM + TFCN	Clustering + Deep Learning	DOA, PA, PW, RF, TOA	Real	10	Partial
Li C. et al., 2025 [25]	China	CBRNN (CNN + BiRNN)	Deep Learning	PA, PW, RF, TOA	Synthetic	Not specified	YES
Çağlan Ş. et al., 2025 [26]	Türkiye	OPTICS and *k*-means	Clustering	AOA, PA, PW, RF, TOA	Synthetic	Multiple	Limited

**Table 2:** Decision rules for the data particle division process (source: own elaboration).

Condition for p*_A_*	Condition for p*_B_*	Decision
*∂_A_* < *ε*	*∂_B_* < *ε*	Data particle **p** is considered a final data particle
*∂_A_* < *ε*	*∂_B_* ≥ *ε*	Data particle **p***_A_* is final, data particle **p***_B_* is further divided
*∂_A_* ≥ *ε*	*∂_B_* < *ε*	Data particle **p***_B_* is final, data particle **p***_A_* is further divided
*∂_A_* ≥ *ε*	*∂_B_* ≥ *ε*	Both data particles **p***_A_* and **p***_B_* are further divided

**Table 3:** Parametrization of clustering algorithms (source: own elaboration).

Algorithm	Parameter	Value
*k*-means	*k*	4
DBSCAN	*ε*	0.25
	*minPts*	10
AGNES	*K*	4
	*linkage*	ward
HDBSCAN	*minSamples*	10
	*min_cluster_size*	50

**Table 4:** Evaluation metrics used in clustering assessments (source: own elaboration based on [36,37]).

Metric	Description
Silhouette Score	Measures cohesion and separation of clusters
Dunn Index	Ratio of minimum inter-cluster distance to maximum intra-cluster distance
Execution Time [s]	Total time required to perform clustering

**Table 5:** Comparison of clustering results and data processing times obtained by the particular methods (source: own elaboration).

Algorithm					
Metric	NHyp2GD	DBSCAN	*k*-means	AGNES	HDBSCAN
Silhouette Score	0.5128	0.5107	**0.5757**	0.5267	0.5101
Dunn Index	0.1844	**0.6628**	0.0035	0.0064	0.2945
Execution Time	**0.0118**	0.0274	1.8195	0.0594	0.0256

## Data Availability

The authors do not have permission to proliferate data.

## Abbreviations

The following abbreviations are used in this manuscript:

AMP	Amplitude
AOA/DOA	Angle of Arrival/Direction of Arrival
BiRNN/BRNN	Bidirectional Recurrent Neural Network
CBRNN	Convolutional Bidirectional Recurrent Neural Network
CNN	Convolutional Neural Network
DL	Deep Learning
DTOA	Difference of Time of Arrival
ELINT	Electronic Intelligence
GRU	Gated Recurrent Unit
JADO	Joint All-Domain Operations
KDE-KLD-TM	Kernel Density Estimation Kullback–Leibler Divergence Template Matching
mAP50	Mean Average Precision at IoU = 0.5
MDO	Multi-Domain Operations
ML	Machine Learning
PA	Pulse Amplitude
PDW	Pulse Description Word
PRI	Pulse Repetition Interval
PW	Pulse Width
RF/CF	Radio Frequency/Carrier Frequency
RNN	Recurrent Neural Network
SEI	Specific Emitter Identification
SOLOv2	Segmenting Objects by Locations v2
TFCN	Time-Frequency Convolutional Network
TOA	Time of Arrival
YOLO	You Only Look Once

## References

[1] Kacmarik O., Vasicek R. (2024). The Multi-Domain Approach to Military Operations and its Challenges to Intelligence and Intelligence, Surveillance, Reconnaissance. Proceedings of the 4th International Scientific Conference "Challenges to National Defence in Contemporary Geopolitical Situation" (CNDCGS’2024).

[2] Chauhan D., Kagathara H., Mewada H., Patel S., Kavaiya S., Barb G. (2025). Nation’s Defense: A Comprehensive Review of Anti-Drone Systems and Strategies. IEEE Access.

[3] Chaari M.Z. (2025). Analysis of the Power of Drones and Limitations of the Anti-Drone Solutions on the Russian-Ukrainian Battlefield. Sec. Def. Quart..

[4] Yu A., Kolotylo I., Hashim H.A., Eltoukhy A.E.E. (2025). Electronic Warfare Cyberattacks, Countermeasures, and Modern Defensive Strategies of UAV Avionics: A Survey. IEEE Access.

[5] Semenyuk L., Kurmashev I., Lupidi A., Alyoshin D., Kurmasheva L., Cantelli-Forti A. (2025). Advances in UAV Detection: Integrating Multi-Sensor Systems and AI for Enhanced Accuracy and Efficiency. Int. J. Crit. Infrastruct. Prot..

[6] Tang Z., Ma H., Qu Y., Mao X. (2025). UAV Detection with Passive Radar: Algorithms, Applications, and Challenges. Drones.

[7] Gautam V., Shishodia V. (2022). The E-Intelligence System. arXiv.

[8] Rybak Ł., Dudczyk J. (2019). Increasing the Information Superiority on the Modern Battlefield Through the Use of Virtual Reality Systems. Sec. Def. Q..

[9] Banasiak K. (2023). Selected Aspects of Measurement Data Processing in Electronic Warfare Devices. Bull. Mil. Univ. Tech..

[10] Dudczyk J., Rybak Ł. (2023). Application of Data Particle Geometrical Divide Algorithms in the Process of Radar Signal Recognition. Sensors.

[11] Banasiak K. (2014). Wykrywanie sygnałów impulsowych w złożonych warunkach ich akwizycji. Pomiary Autom. Kontrola (PAK).

[12] Chao W., Weisong L., Xueqiong L., Xiang W., Zhitao H. (2022). A New Radar Signal Multiparameter-Based Deinterleaving Method. arXiv.

[13] Rybak Ł., Dudczyk J., Mazur P. (2024). Algorytmy sztucznej inteligencji w przetwarzaniu danych rozpoznania radioelektronicznego ELINT. Przegląd Telekomun.—Wiadomości Telekomun..

[14] Pedregosa F., Varoquaux G., Gramfort A., Michel V., Thirion B., Grisel O., Blondel M., Prettenhofer P., Weiss R., Dubourg V. (2011). Scikit-learn: Machine Learning in Python. J. Mach. Learn. Res..

[15] Rybak Ł., Dudczyk J. (2020). A Geometrical Divide of Data Particle in Gravitational Classification of Moons and Circles Data Sets. Entropy.

[16] Rybak Ł., Dudczyk J. (2021). Variant of Data Particle Geometrical Divide for Imbalanced Data Sets Classification by the Example of Occupancy Detection. Appl. Sci..

[17] Rybak Ł. (2022). Geometryczny Podział Cząstki Danych w Klasyfikacji Wielowymiarowych Zbiorów Danych. Doctoral Thesis.

[18] Rybak Ł., Dudczyk J. (2025). Impact of Data Particle Divide Depth Level on the Effectiveness of Hypergeometrical Divide Classifier. Bull. Pol. Acad. Sci. Tech. Sci..

[19] Kocamış M.B., Orduyılmaz A., Taşcıoğlu S. (2024). Object detection based deinterleaving of radar signals using deep learning for cognitive EW. Sig. Img. Vid. Process.

[20] Liu H., Wang L., Wang G. (2024). Emitter Signal Deinterleaving Based on Single PDW with Modulation-Hypothesis-Augmented Transformer. Remote Sens..

[21] Chen T., Guo X., Li J. (2024). Radar Signal Sorting Method with Mimetic Image Mapping Based on Antenna Scan Pattern via SOLOv2 Network. Remote Sens..

[22] Gunn E., Hosford A., Mannion D., Williams J., Chhabra V., Nockles V. (2025). Radar Pulse Deinterleaving with Transformer Based Deep Metric Learning. arXiv.

[23] Campello R.J., Moulavi D., Sander J., Pei J., Tseng V.S., Cao L., Motoda H., Xu G. (2013). Density-based Clustering Based on Hierarchical Density Estimates. Advances in Knowledge Discovery and Data Mining.

[24] Zhang J., Wang B., Han X., Zhao M., Liang Z., Chen X., Liu Q. (2025). A multi-radar emitter sorting and recognition method based on hierarchical clustering and TFCN. Digit. Signal Process..

[25] Li C., Zheng J., Chen R., Liu H. (2025). A framework for radar signal deinterleaving and parameter estimation based on split pulse features extracted by deep learning. Expert Syst. Appl..

[26] Çağlan Ş., Değirmenci A., Çankaya İ. (2025). A New Deinterleaving Approach Based on Clustering and PRI Type Recognition. J. Polytech..

[27] Reddy R., Sinha S. (2025). State-of-the-Art Review: Electronic Warfare Against Radar Systems. IEEE Access.

[28] Wright W.E. (1977). Gravitational clustering. Pattern Recognit..

[29] Liu C., Wang W., Tu G., Xiang Y., Wang S., Lv F. (2017). A new Centroid-Based Classification Model for Text Categorization. Knowl. Based Syst..

[30] Olszewski J., Mazur P., Rybak Ł., Dudczyk J. (2025). Właściwości algorytmu hipergeometrycznego podziału cząstki danych w aspekcie przetwarzania danych wywiadu elektronicznego. Przegląd Telekomun.—Wiadomości Telekomun..

[31] Peng L., Chen Y., Yang B., Chen Z. (2005). A Novel Classification Method Based on Data Gravitation. Proceedings of the 2005 International Conference on Neural Networks and Brain (ICNN&B’05), Beijing, China, 13–15 October 2005.

[32] MacQueen J. (1967). Some Methods for Classification and Analysis of Multivariate Observations. Proceedings of the 5th Berkeley Symposium on Mathematical Statistics and Probability.

[33] Ester M., Kriegel H.P., Sander J., Xu X. A Density-Based Algorithm for Discovering Clusters in Large Spatial Databases with Noise. Proceedings of the 2nd International Conference on Knowledge Discovery and Data Mining (KDD-96).

[34] Amalina D.N., Fauzan A. (2024). A Hierarchical Density-Based Spatial Clustering of Applications with Noise (HDBSCAN) Approach for Identifying Potential Villages in Buleleng Regency. Knowl. Eng. Data Sci..

[35] Jain A.K., Murty M.N., Flynn P.J. (1999). Data clustering: A review. ACM Comput. Surv..

[36] Rousseeuw P.J. (1987). Silhouettes: A Graphical Aid to the Interpretation and Validation of Cluster Analysis. J. Comput. Appl. Math..

[37] Dunn J.C. (1974). Well-Separated Clusters and Optimal Fuzzy Partitions. J. Cybern..

